# Evaluation of Cardiotoxicity in Lymphoproliferative Patients Treated With Anthracycline and Bruton's Tyrosine Kinase Inhibitor-Based Regimens

**DOI:** 10.7759/cureus.95077

**Published:** 2025-10-21

**Authors:** Andrew Bonsu

**Affiliations:** 1 General Surgery, New Cross Hospital, Wolverhampton, GBR

**Keywords:** anthracyclines, bruton tyrosine kinase inhibitor, cardio-oncology, cardiotoxic agents, lymphoproliferative malignancy

## Abstract

Background: The treatment of lymphoproliferative disorders has often included a range of cardiotoxic chemotherapy-focused regimens, in particular Bruton’s tyrosine kinase inhibitors (BTKi) and anthracyclines. Despite these agents being commonly used in clinical practice, the specific process of cardiotoxicity and the prevention of the subsequent cardiac-based pathologies, including atrial fibrillation and hypertension, are increasing points of research. Many hematology-oncology tertiary centers incorporate these agents into regular treatment regimens for patients. A retrospective study from a large patient cohort was pivotal in highlighting the specific cardiovascular risks involved with the applications of these agents. Adding to this, the increased scope of evidence shows an estimated 2-4% of all patients treated with anthracyclines developing left ventricular dysfunction after nine years, with six to eight people per 1000 persons treated with BTKis developing ventricular arrhythmias and/or sudden cardiac death.

Materials and methods: The study population comprised 389 patients included in the patient study pool from January 2018 to June 2023. The medical histories of all patients were reviewed to determine the incidence of specific cardiac-related pathologies such as atrial fibrillation, ventricular arrhythmias, cardiac arrest/sudden death, heart failure, and hypertension, both pre- and post-chemotherapy administration. The patients were divided into an anthracycline and a BTKi cohort, of which 2% of the anthracycline cohort presented with either atrial fibrillation, ventricular tachycardia, or heart failure, while 6% of the BTKi cohort presented with either atrial fibrillation, hypertension, or heart failure.

Results: Cardiotoxic effects were noted in a shorter timespan post-anthracycline administration (0-91 days) as compared to post-BTKi administration (245-1539 days).

Conclusions: Our study identifies a significant cardiotoxic burden following chemotherapy initiation, which is most pronounced with the application of anthracyclines as compared to BTKis. This evidence may benefit patients with lymphoproliferative disorders through prospective cardiovascular assessment and monitoring before, during, and after treatment.

## Introduction

Through the advances of modern medicine, significant progress in the early identification and detection of lymphoproliferative disorders has enabled a noted decline in both morbidity and mortality rates [1]. Alongside identification and detection, the rise of chemotherapeutic agents has considerably contributed to the improvement of survival rates. Two key examples of these agents are the anthracycline drug family (i.e., daunorubicin, doxorubicin, and epirubicin) and Bruton’s tyrosine kinase inhibitors (BTKi) [2-6].

Anthracyclines have been among the most potent antineoplastic medication classes in oncology treatment, partly due to their mechanism of action targeting topoisomerase II (TOPO-II) [7-8]. This enzyme works to cleave strands of DNA and yield double-strand breaks. Anthracyclines bind to TOPO-II DNA complexes, inhibiting both DNA replication and gene transcription. Additionally, they generate reactive oxygen species (ROS), causing DNA damage to cancerous cells [9-10].

Alongside anthracyclines are also BTKis. These potent therapeutic agents primarily invoke their cytotoxic effects by binding to a specific protein kinase (Bruton’s tyrosine kinase) critical in the B-cell signalling cascades that can lead to the development of many hematological malignancies [11-13]. BTKis work to interfere with the kinase’s ability to bind with key substrates and thus induce cytotoxic effects.

Despite both anthracyclines and BTKis displaying effective anticancer properties, their unintended side effects have been known to lead to unforeseen cardiotoxicity-related events, most commonly atrial fibrillation, ventricular arrhythmias, sudden death, hypertension, and heart failure [14-16].

Concerning the mechanisms of anthracycline-induced cardiotoxicity, a multitude of molecular pathways have been highlighted as implicated causes, yet there is an incomplete understanding of the exact underlying cause. Two commonly accepted mechanisms are that anthracycline-induced ROS and drug-induced inhibition of TOPO-IIβ within cardiac muscle cells are key factors in cardiotoxicity, leading to cell degradation and death [14,17].

Similarly, the definitive mechanism of how cardiotoxicity presents is still unknown in BTKis. Current literature supports the view of C-terminal Src kinase (CSK) and phosphoinositide-3 kinase (PI3K) inhibition, as examples of off-target kinase inhibition, playing key roles in the development of several adverse cardiovascular events [18]. Studies show that CSK is predominantly present in atrial cells, and hence, inhibition of this enzyme has been observed to increase the incidence of atrial fibrillation [19]. Additionally, literature has discussed the effects of PI3K inhibition, which increases the susceptibility to ventricular arrhythmias and hypertension [18,20].

Although there is a present knowledge gap regarding the mechanisms of cardiotoxic events with the use of these chemotherapeutic agents, the point remains that these agents are prone to provoking cardiotoxic events and particularly exacerbating clinical presentations of such events in patients with a pre-existing cardiovascular disease history. As these are commonly applied in a multitude of chemotherapy regimens used to treat hematological malignancies, it is important to understand both the burden of pre-existing cardiovascular disease risk factors and to establish the true impact of these treatment regimens on patients.

This work was initially presented as a poster presentation at the 2024 EHA-BSH Conference in Liverpool. Further review of the data revealed that 6% of BTKi patients, instead of the initially reported 2%, had presented with cardiotoxic events, and this has been amended accordingly in this paper.

## Materials and methods

Patients were identified from a lymphoproliferative disorder patient database in a tertiary cancer center. All patients with an active diagnosis of a lymphoproliferative disorder, treated with either an anthracycline-based or BTKi-based regimen between January 2018 and June 2023, were included and divided into two separate cohorts for comparative analysis. Patient demographics and the clinical diagnosis were captured by medical record review. Any comorbid conditions known to be associated with increased cardiovascular disease risk, including hypertension, diabetes mellitus, and hypercholesterolemia, were identified and logged using the center’s medical record system. The treatment cycles of each patient were reviewed to log the time elapsed before any cardiotoxic event occurred, along with the specific type of event. The purpose was to identify consistent trends regarding chemotherapy regimens and cardiotoxic events. In addition to this, if a patient required treatment dose reductions/alternative treatment regimens due to the presentation of a cardiotoxic event or if additional medications were required for symptom management, these were also reviewed and noted as part of the study.

The patient cohort selected for this retrospective study required no review board approval because the information collected on post-treatment side effects was encrypted in medical records, ensuring no patient-identifiable details were revealed at any stage.

The exclusion criteria for the patients selected for this retrospective study consisted of the following: patients who did not have a confirmed diagnosis of a lymphoproliferative disorder, patients managed with an alternative form of chemotherapy outside of anthracyclines and/or BTKis, and lastly, patients who were deceased prior to commencing any form of anthracycline-based or BTKi-based chemotherapy regimens.

## Results

The study population had 389 patients, 300 of whom were treated with anthracyclines, and the remaining 89 were treated with BTKis.

Anthracycline cohort

Regarding the anthracycline cohort, 57% were male with a median age of 56, while the remaining 43% were female with a median age of 57. Within this cohort, 27% had Hodgkin’s lymphoma, while 58% had non-Hodgkin’s lymphoma. At the time of the diagnosis of the lymphoproliferative disorder, 15% of patients had a background of hypertension (9% of male and 6% of female), 6% had a background of type 2 diabetes mellitus (4% of male and 2% of female), and 3% had a background of hypercholesterolemia (2% of male and 1% of female), as highlighted in Table 1.

**Table 1:** Anthracycline demographics ^A ^Female patient presenting with heart failure, requiring a switch to RGCVP. ^B ^Female patient presenting with heart failure, requiring a switch to azacitidine + venetoclax. ^C ^Male patient presenting with atrial fibrillation, requiring cessation of chemotherapy. ^D ^Male patient presenting with ventricular tachycardia, requiring treatment with bisoprolol. ^1 ^Male patient presenting with asymptomatic sinus tachycardia. ^2 ^Male patient presenting with asymptomatic sinus tachycardia. ^3 ^Male patient presenting with asymptomatic sinus tachycardia. ^4 ^Male patient presenting with sinus bradycardia with a family history of long QT syndrome. This patient also had an episode of ventricular arrhythmia, which was managed with bisoprolol. ^5 ^Male patient presenting with symptomatic tachycardia, requiring treatment with ivabradine. ^6 ^Female patient presenting with long QT syndrome, requiring switch to azacitidine. ^7 ^Female patient presenting with sinus tachycardia. M: male, F: female, RGCVP: rituximab, gemcitabine, cyclophosphamide, vincristine, prednisolone

Anthracycline demographics	Overall (n = 300) (%)
Sex	
Male	171 (57)
Female	129 (43)
Age	
Male median age	56 (32)
Female median age	57 (33)
Malignancy	
Hodgkin lymphoma (male)	48 (16)
Hodgkin lymphoma (female)	33 (11)
Non-Hodgkin lymphoma (male)	109 (33)
Non-Hodgkin lymphoma (female)	76 (25)
Chronic lymphocytic leukemia	0 for both M+F
Comorbidities	
Hypertension (male)	25 (9)
Hypertension (female)	21 (7)
Diabetes mellitus (male)	13 (4)
Diabetes mellitus (female)	5 (2)
Hypercholesterolemia (male)	6 (2)
Hypercholesterolemia (female)	4 (1)
Cardiotoxicity side effects	
Atrial fibrillation (male)	1 (1)
Atrial fibrillation (female)	0
Ventricular arrhythmia (male)	1
Ventricular arrhythmia (female)	0
Cardiac arrest/sudden cardiac death	0 for both M+F
Hypertension	0 for both M+F
Heart failure (male)	0
Heart failure (female)	2
Other cardiac-related side effects (male)	5^1-5 ^(2)
Other cardiac-related side effects (female)	2^ 6, 7 ^(1)
Time from start of therapy to side effects	
Male	80 days (68-91)
Female	32 days (0-63)
Outcome of side effects	
Dose reduction	0 for both M+F
Anthracycline withheld/alternative treatment (male)	1^D ^(1)
Anthracycline withheld/alternative treatment (female)	2^A, B^ (1)
Additional medications for symptom management (male)	2^C, D^ (1)
Additional medications for symptom management (female)	0

Treatment review of this cohort revealed 2% of patients presenting with cardiotoxic events. Some of the presentations include a patient treated with ABVD who had presented with heart failure shortly after the first cycle, and despite the first cycle involving doxorubicin, the severity of the heart failure meant that the patient was eventually switched to RGCVP three weeks after initial presentation. Another patient had presented with new-onset atrial fibrillation soon after initiating treatment. This was initially treated with anticoagulation therapy, and chemotherapy persisted until the fifth cycle, when chemotherapy was ceased due to the declining performance status of the patient.

Now, despite only 2% of patients presenting with cardiotoxic events, 2% of patients separated from this cardiotoxic event group had presented with tachycardia, bradycardia, or long QT syndrome. Most patients in the tachycardia group had a singular asymptomatic sinus episode that had resolved spontaneously; however, a patient had presented with a queried sinus tachycardia after the fourth treatment cycle, which was further complicated by the patient additionally presenting with a left arm DVT. Another patient had presented with symptomatic tachycardia during their third treatment cycle, with queries that the presentation may be postural orthostatic tachycardia syndrome. The patient was subsequently treated with Ivabradine; no further episodes were noted. For the long QT syndrome patient, this presentation came shortly after their first treatment cycle. After further review, the patient was switched to azacitidine, with no further long QT syndrome events noted. The final patient had presented initially with ventricular arrhythmia after the third treatment cycle and was managed with bisoprolol. After the fourth treatment cycle, the same patient went on to present with sinus bradycardia. This specific occurrence was notably significant, as the patient had a long QT syndrome family background (Table 2).

**Table 2:** Breakdown of anthracycline side effect outcomes DVT: deep vein thrombosis, ECG: electrocardiogram, RGCVP: rituximab, gemcitabine, cyclophosphamide, vincristine, and prednisolone

Note number	Details of side effects
^A^	Female patient with heart failure on 07/11/2022, doxorubicin withheld on 09/11/2022, even with 75% doxorubicin dosing from Cycle 1, and treatment switched to RGCVP from 28/11/2022.
^B^	Female patient with heart failure on 08/02/2022, on Cycle 1, the patient reacted and developed heart failure + cytokine syndrome. DA was stopped, and the chemotherapy regimen was converted to azacitidine + venetoclax on 17/02/2022.
^C^	Male patient with atrial fibrillation that was treated with Clexane and chemotherapy, ceased on 30/09/2021 after Cycle 5 due to failing performance status.
^D^	Male patient with ventricular arrhythmia (tachycardia) after Cycle 3 on 24/11/2021 and was managed with bisoprolol. Treatment was postponed from 24/11/2021 until 18/12/2021.
^1^	Male asymptomatic sinus tachycardia on 15/04/2021 – spontaneously resolving.
^2^	Male asymptomatic sinus tachycardia on 23/02/2021 and resolved on 02/03/2021 spontaneously.
^3^	Male asymptomatic sinus tachycardia on 02/06/2021 and resolved spontaneously with no recurrence.
^4^	Male sinus bradycardia in a patient with a family background of long QT syndrome after Cycle 4 on 20/12/2021. This patient also had a ventricular arrhythmia on 24/11/2021, which was managed with bisoprolol.
^5^	Male symptomatic tachycardia on 13/05/2022 during Cycle 3 treated with ivabradine, as it was suggested that the patient might have postural orthostatic tachycardia syndrome.
^6^	Female long QT syndrome on ECG and patient was managed via azacitidine – alternative treatment.
^7^	Female sinus tachycardia with symptoms queried on 30/06/2021 after Cycle 4, as the patient also had a left arm DVT.

The review of the patients within the anthracycline cohort has provided strengthened evidence regarding the relationship between cardiotoxic presentations and the implementation of anthracyclines.

BTKi cohort

For the BTKi cohort, 57% were male with a median age of 71, while the remaining 43% were female with a median age of 71. Within this cohort, 25% had a background of non-Hodgkin’s lymphoma and 75% had a background of chronic lymphocytic leukemia. At the point of being diagnosed with a lymphoproliferative disorder, 28% of patients had a background of hypertension (18% of male and 10% of female), 12% had a background of type 2 diabetes mellitus (6% of male and 6% of female), and 8% had a background of hypercholesterolemia (7% of male and 1% of female) (Table 3).

**Table 3:** BTKi demographics ^1 ^Male patient presenting with atrial fibrillation and hypertension, requiring temporary treatment cessation alongside symptom management with both doxazosin and amlodipine. ^2 ^Male patient presenting with atrial flutter, requiring treatment switch from ibrutinib to zanubrutinib. ^3 ^Male patient presenting with hypertension, requiring temporary treatment cessation and symptom management with amlodipine. ^4 ^Female patient presenting with atrial fibrillation, requiring switch of treatment to venetoclax and rituximab. ^5 ^Female patient presenting with hypertension, requiring switch from ibrutinib to zanabrutinib and symptom management with amlodipine. ^6 ^Female patient presenting with hypertension, requiring the use of losartan, which was uptitrated to help manage symptoms. BTKi: Bruton’s tyrosine kinase inhibitor

BTKi demographics	Overall (n = 89) (%)
Sex	
Male	51 (57)
Female	38 (43)
Age	
Male median age	71 (17)
Female median age	71 (12)
Malignancy	
Hodgkin lymphoma	0 for both M+F
Non-Hodgkin lymphoma (male)	14 (16)
Non-Hodgkin lymphoma (female)	8 (9)
Chronic lymphocytic leukemia (male)	37 (42)
Chronic lymphocytic leukemia (female)	30 (34)
Comorbidities	
Hypertension (male)	16 (18)
Hypertension (female)	9 (10)
Diabetes mellitus (male)	5 (6)
Diabetes mellitus (female)	5 (6)
Hypercholesterolemia (male)	6 (7)
Hypercholesterolemia (female)	1 (1)
Cardiotoxicity side effects	
Atrial fibrillation (male)	1^1^ (1)
Atrial fibrillation (female)	1^4 ^(1)
Ventricular arrhythmia	0 for both M+F
Cardiac arrest/sudden cardiac death	0 for both M+F
Hypertension (male)	2^1, 3^ (2)
Hypertension (female)	2^5, 6^ (2)
Heart failure	0 for both M+F
Other cardiac-related side effects (male)	1^2^ (1)
Other cardiac-related side effects (female)	0
Average time from the start of therapy to side effects	
Male	892 days (245-1539)
Female	408 days (294-495)
Outcome of side effects	
Dose reduction	0 for both M+F
BTKi withheld/alternative treatment (male)	2^1, 3^ (2)
BTKi withheld/alternative treatment (female)	2^4, 5^ (2)
Additional medications for symptom management (male)	2^1, 3^ (2)
Additional medications for symptom management (female)	2^6^ (2)

As compared to the anthracycline cohort, 6% of patients presented with cardiotoxic events, which comprised atrial fibrillation and hypertension. Regarding the hypertension group, the patients who had presented with these cardiotoxic events were managed with different approaches. The BTKi treatment for one patient was withheld for two months while the hypertension was being treated with an antihypertensive in the interlude. The BTKi treatment was substituted for another BTKi in the case of a separate patient due to excessive bruising alongside the unresolved hypertension, despite the addition of an antihypertensive used for this patient. A separate patient had maintained their original BTKi, despite presenting with hypertension, as compared to the previous two patients, and instead had an antihypertensive implemented, which was subsequently uptitrated.

For the atrial fibrillation group, the BTKi treatment for one of the patients had been ceased due to the presentation of atrial fibrillation and subsequently substituted with venetoclax and rituximab. The final patient was a unique case in that both presentations of atrial fibrillation and hypertension were observed during their treatment cycles. The BTKi treatment for this patient was withheld for 2.5 months while the patient was managed with two antihypertensives in the interlude period.

One separate patient from the atrial fibrillation and hypertension patient groups had an acute episode of atrial flutter, which caused the withholding of their treatment BTKi for 1.5 months initially. The BTKi treatment was then restarted and continued for the following four months. During this period, the atrial flutter returned, so the treatment BTKi was substituted with an alternative BTKi (Table 4). The results provided for the BTKi cohort further strengthen the knowledge concerning the relationship between BTKis and cardiotoxic events.

**Table 4:** Breakdown of BTKi side effect outcomes BTKi: Bruton’s tyrosine kinase inhibitors, AF: atrial fibrillation, HTN: hypertension, OD: once daily

Note number	Details of side effects
^1^	One AF + HTN male patient presented with ibrutinib use, which was withheld from 25/04/2022 until 04/07/2022, while also given doxazosin and amlodipine at 10 mg.
^2^	One male atrial flutter was present on 19/10/2022 and suspended until 07/12/2022. The flutter returned on 12/04/2023, so ibrutinib was withheld and substituted with zanubrutinib.
^3^	One male HTN patient presented on 15/08/2022, and so acalabrutinib was withheld from 15/08/2022 to 09/11/2022, and 10 mg amlodipine was prescribed.
^4^	A female patient presents with AF and ibrutinib use. Ibrutinib ceased on 19/10/2021 due to AF, and treatment was switched to venetoclax and rituximab in March 2023.
^5^	HTN female patient 1 presents with ibrutinib use, ibrutinib changed to zanubrutinib in March 2023 due to excessive bruising in March 2023 and 10 mg amlodipine was prescribed.
^6^	HTN female patient 2 presents with ibrutinib use, ibrutinib maintained at the same dose, but losartan was added on 30/03/2022 due to HTN secondary to ibrutinib and increased to 75 mg OD.

Average time from the start of therapy to the presentation of cardiotoxic events presentation

Comparative analysis of the two chemotherapy cohorts revealed that the anthracycline cohort experienced cardiotoxic events more quickly than the BTKi cohort (91 days for the anthracycline cohort vs. 1539 days for the BTKi cohort). In addition, across both cohorts, it was found that female patients, on average, presented much earlier with cardiotoxic events as compared to the male patients, whereby the anthracycline cohort had an average of 32 days for female patients vs. 80 days for male patients. The BTKi cohort had female patients presenting on average after 408 days, compared to 892 days for male patients.

## Discussion

This retrospective study is key because it provides several clinically vital results regarding the use of anthracyclines and BTKis as chemotherapeutic agents for lymphoproliferative disorders, as well as the increased incidence of cardiotoxic events associated with these agents. Through this study, evidence has shown that the overall incidence of cardiotoxic events was increased mildly when anthracyclines and/or BTKis are used as treatment options.

**Figure 1:**
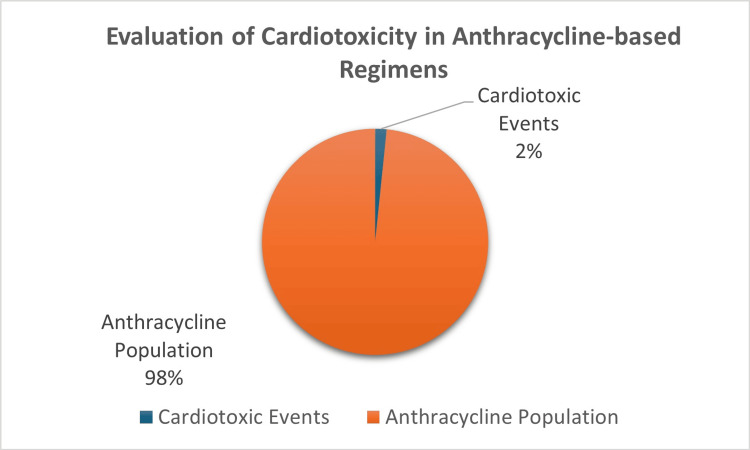
Pie chart illustration showing the anthracycline population presenting with cardiotoxic effects

About the anthracycline cohort, 2% of patients had presented with a cardiotoxic event as specified in the study criteria (Figure 1). In this cohort, patients presented with either atrial fibrillation or ventricular arrhythmia, with atrial fibrillation being more common, consistent with established literature on the increased incidence of atrial fibrillation when using anthracyclines [21-23]. In addition to this, 2% of patients separately presented with arrhythmia-related cardiotoxic events. This may suggest that, despite anthracyclines having a potentially broad range of cardiotoxic effects, there is a particular focus on their impact on natural heart rhythms and their tendency to cause arrhythmias in patients.

Analysis of this cohort revealed an increased incidence of male patients presenting with cardiotoxic events as compared to female patients (two male patients to one female patient). In addition, a greater incidence of male patients in the arrhythmia-related cardiotoxic event group was found as compared to female patients (five male patients to two female patients). However, a key point to consider is that the average time of clinical presentation of the cardiotoxic event in female patients was significantly shorter than in male patients. Given these results, it may be useful for future investigations to consider whether the sex of a lymphoproliferative patient has any considerable effect on the incidence of cardiotoxic events when using anthracyclines as treatment.

**Figure 2:**
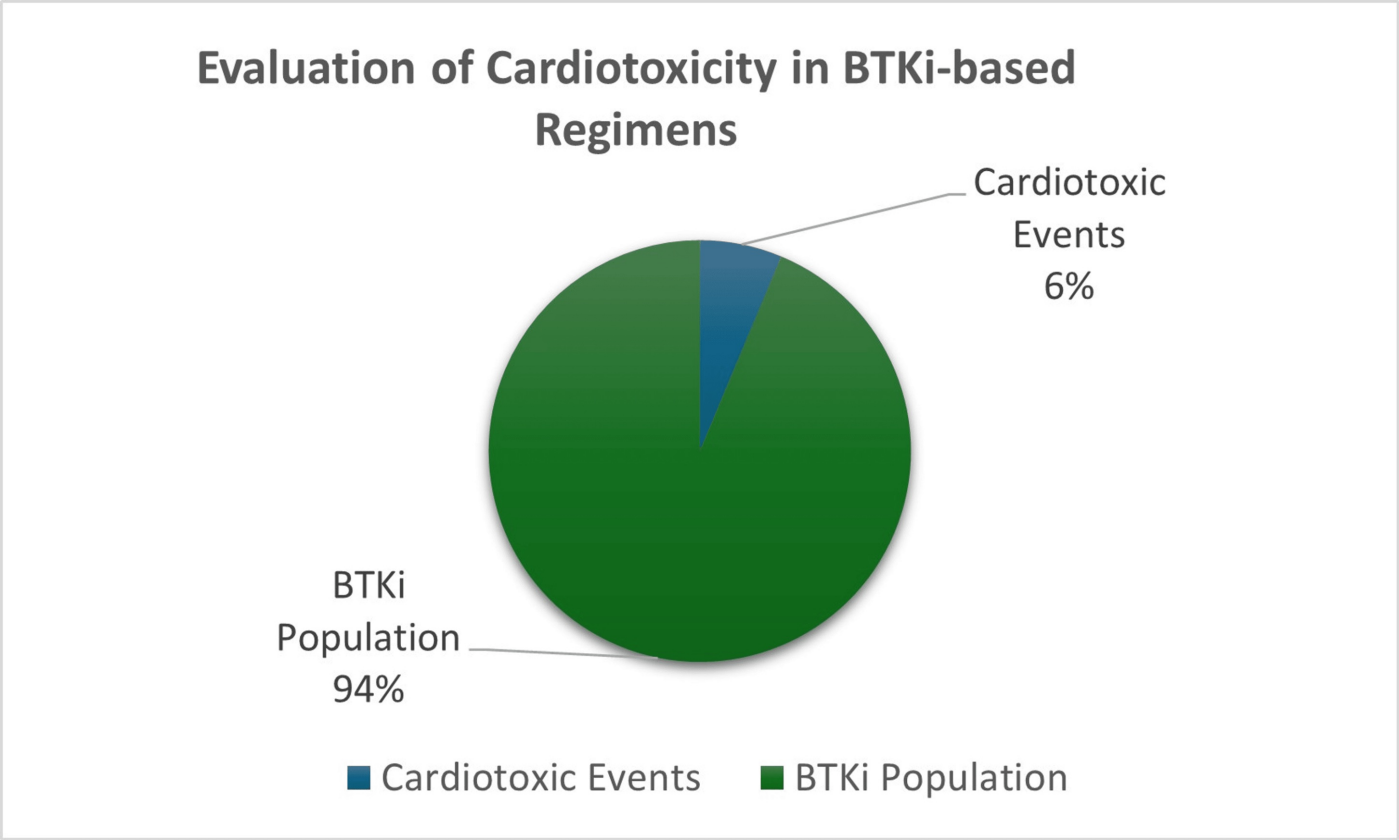
Pie chart illustration showing the BTKi population presenting with cardiotoxic effects BTKi: Bruton’s tyrosine kinase inhibitor

Despite the BTKi cohort being significantly smaller in sample size than the anthracycline cohort, 6% of patients in this cohort had presented with noted cardiotoxic events, which were either atrial fibrillation or hypertension (Figure 2). In contrast to the anthracycline cohort, hypertension served as the main cardiotoxic event observed in the BTKi cohort. Four percent of patients had presented with hypertension when treated with BTKis, whereas 2% of patients presented with atrial fibrillation [23].

In addition, a lower incidence of arrhythmia-related cardiotoxic events was observed in patients with BTKis than in the anthracycline cohort. A single male patient presented with atrial flutter while treated with a BTKi, but the atrial flutter ceased once the BTKi treatment was substituted. Analysis of the BTKi cohort revealed that ibrutinib was the initial BTKi for many patients and that substituting it with a different BTKi alleviated the presentation of cardiotoxic events. With these findings presented in this study, future studies are crucial to better understand the mechanisms of BTKi, particularly newer generation types compared to ibrutinib, to assess susceptibility to cardiotoxic events.

Similarly, as observed in the anthracycline cohort, the average time to clinical presentation of cardiotoxic events in female patients was shorter than in male patients (408 days vs. 892 days). Acknowledging this, the first clinical presentation of a male patient occurred after 245 days of treatment, compared to 294 days for a female patient. Nevertheless, the similar findings observed in the BTKi cohort regarding sex and the average time of initial symptom presentation may be a scope of interest for in-depth consideration in future studies.

The knowledge regarding the cardio-oncological impact of anthracyclines and BTKis is not unique to this study, as previous evidence has highlighted baseline information about these chemotherapeutic agents. The European Society of Cardiology (ESC) has launched a guideline in collaboration with the European Haematology Association (EHA), focusing on the assessment methods applied to patients before treatment with anthracyclines and BTKis. This guideline advises all patients to undergo a cardiovascular toxicity risk assessment, which includes a comprehensive history and examination, baseline ECG, baseline cardiac serum biomarkers such as natriuretic peptides and cardiac troponins, cardiac imaging via an echocardiogram, and cardiopulmonary exercise testing [24-25].

Limitations

This study carries several limitations. This was a single-center retrospective study in which the diagnosis of the lymphoproliferative disorder was acknowledged via clinical letters and the incorporation of a medical database. No data points were collected during the follow-up regarding the presence of cardiomyopathies, notable metabolic derangements, or electrolyte abnormalities in the patient cohort, which might have predisposed them to cardiotoxic events. Additionally, the exclusion criteria did not consider loss due to lack of follow-up, which may have impacted the final sample size for analysis. Furthermore, the sample size of the two patient cohorts is significantly skewed towards that of the anthracycline cohort, with 300 patients under this remit compared to 89 for the BTKi cohort, which may cast doubt on how reliably the data can be compared.

Because several chemotherapy regimens were incorporated into the anthracycline cohort using different anthracyclines, it is unclear which anthracycline is more prone to exerting cardiotoxic effects compared to the BTKi cohort. As such, future studies would be beneficial in gaining clarity regarding the tendencies of specific anthracyclines to cause cardiotoxic events.

In the BTKi cohort, many patients were treated with ibrutinib, and any observed cardiotoxic events were managed by adding an antihypertensive, substituting ibrutinib with either an alternative BTKi or another treatment regimen, or temporarily ceasing ibrutinib. Despite this, future projects are necessary to compare the cardiotoxic potential of newer-generation BTKis as compared to the established ibrutinib.

## Conclusions

The data yielded from this study show that anthracycline and/or BTKi use is associated with an increased risk of cardiotoxic events, including arrhythmic episodes, ventricular failure, and hypertension observed in lymphoproliferative patients. This risk is significantly pronounced among those treated with ibrutinib, but clarity is needed regarding which specific anthracycline(s) may cause an increased incidence. Studies for the future are needed to assess the treatment algorithms helping to reduce the risk of cardiotoxic events in lymphoproliferative disorders requiring chemotherapeutic agents.

With the knowledge gained about anthracyclines and BTKis, the field known as cardio-oncology continues to expand as an established subspecialty. With detailed, comprehensive guidelines similar to those provided by the ESC, there is further insight into close cardiac monitoring and symptom awareness in hemato-oncology patients receiving potentially cardiotoxic treatment regimens. This last point is crucial to consider, as it is pivotal in ensuring the cardioprotection of these patients undergoing such treatments. Improved screening tests, closer monitoring, and the further development of dedicated cardio-oncology clinics will achieve this.
